# ^**1**^H NMR Metabolic Profiling of Biofluids from Rats with Gastric Mucosal Lesion and Electroacupuncture Treatment

**DOI:** 10.1155/2015/801691

**Published:** 2015-06-15

**Authors:** Jingjing Xu, Kian-Kai Cheng, Zongbao Yang, Chao Wang, Guiping Shen, Yadong Wang, Qiong Liu, Jiyang Dong

**Affiliations:** ^1^Department of Electronic Science and Department of Traditional Chinese Medicine, Xiamen University, Xiamen 361005, China; ^2^Department of Bioprocess Engineering and Innovation Centre in Agritechnology, Universiti Teknologi Malaysia, 81310 Johor Bahru, Malaysia; ^3^College of Acupuncture and Moxibustion, Hunan University of Traditional Chinese Medicine, Changsha 410000, China

## Abstract

Gastric mucosal lesion (GML) is a common gastrointestinal disorder with multiple pathogenic mechanisms in clinical practice. In traditional Chinese medicine (TCM), electroacupuncture (EA) treatment has been proven as an effective therapy for GML, although the underlying healing mechanism is not yet clear. Here, we used proton nuclear magnetic resonance- (^1^H NMR-) based metabolomic method to investigate the metabolic perturbation induced by GML and the therapeutic effect of EA treatment on stomach meridian (SM) acupoints. Clear metabolic differences were observed between GML and control groups, and related metabolic pathways were discussed by means of online metabolic network analysis toolbox. By comparing the endogenous metabolites from GML and GML-SM groups, the disturbed pathways were partly recovered towards healthy state via EA treated on SM acupoints. Further comparison of the metabolic variations induced by EA stimulated on SM and the control gallbladder meridian (GM) acupoints showed a quite similar metabolite composition except for increased phenylacetylglycine, 3,4-dihydroxymandelate, and meta-hydroxyphenylacetate and decreased N-methylnicotinamide in urine from rats with EA treated on SM acupoints. The current study showed the potential application of metabolomics in providing further insight into the molecular mechanism of acupuncture.

## 1. Introduction

Gastric mucosal lesion (GML) is a common digestive system disorder which frequently arises from various endogenous and exogenous factors such as stress, smoking, nutritional deficiencies, hydrochloric acid, pepsin,* Helicobacter pylori*, nonsteroidal anti-inflammatory drug (NSAIDs), alcohol, and infection [1]. The therapies of GML in Western medicine focus on symptomatic treatment, and it may be accompanied by unwanted side effects, incomplete remedy and recurrent episodes, and so forth [2]. Acupuncture, one of the elements in traditional Chinese medicine (TCM) practiced for thousands of years in China, has been confirmed to be an effective therapeutic technique for health keeping and disease treatment [3, 4]. Electroacupuncture (EA) is a modification of conventional acupuncture practice, in which EA stimulates acupoint with an electrical current instead of manual manipulations. Multiple studies have showed that EA produces more consistent and reproducible results in both clinical and research settings [5, 6].

In animal studies, acupuncture on stomach meridian (SM) acupoints was shown to have beneficial effects on various gastrointestinal diseases, including irritable bowel syndrome [7, 8], gastroparesis [9], functional dyspepsia [9], constipation [10], and diarrhea [11]. In rats with stress-induced gastric ulcer, EA treatment protects the stomach by thickening gastric mucosal barrier, stabilizing mast cells, and decreasing the gastrin level in gastric mucosa [12]. Furthermore, acupuncture on* Zusanli* points in gastric meridian has been shown to enhance the regularity of gastric myoelectrical activity, accelerate gastric emptying through the vagal pathway, and regulate the content of gastrin, Substance P (SP), epidermal growth factor (EGF), and transforming growth factor-*α* (TGF-*α*) in serum and gastric mucosa [13, 14]. However, the detailed molecular mechanisms of GML and the underlying effects following stimulation of acupuncture points are still unclear.

Here, we aim to use metabolomic approach to investigate the metabolic perturbations induced by GML and EA treatment [15]. As a systemic approach, metabolomics reflects the function of organisms from the end products of the metabolic network to metabolic changes of a complete system caused by interventions in a holistic context [16]. This property agrees with the holistic thinking of TCM suggesting that metabolomics has the potential to facilitate the understanding of the theory behind evidence-based Chinese medicine [17]. In the present study, the biochemical variations of serum and urine from stress- and water-immersion-induced GML rats and EA treatment were investigated by combining high resolution ^1^H NMR spectroscopy with multivariate statistical analysis. This NMR-based metabolomics provides insight into the dysfunction of GML modelling and dissects the mechanism of EA as an effective treatment for GML. The stomach meridian acupoints of “Foot-Yangming” are picked up as functional points to stomach which have been commonly used in human acupuncture to treat gastrointestinal disorders and others [18]. And the gallbladder meridian (GM) acupoints are chosen as control acupoints. The specific and unique biochemical pathways of EA treatment are also identified and discussed. The current results validate the applicability of NMR-based metabolomics in the study of TCM which probe into the establishing of international standards for the TCM modernization.

## 2. Methods and Materials

### 2.1. Animal Handling

Animal care and experimental procedures were approved and performed according to the guidelines of Animal Care and Use Committee of Xiamen University (Permit Number: SCXK 2008-0001). All healthy male rats (150 ± 20 g weight) were housed individually in metabolism cages. The animal room was under controlled condition (temperature, humidity, and 12-hour light-dark cycle) and the animals were provided with food and water* ad libitum*.

Following adaptation for a week, rats were randomly separated into two groups, that is, control and gastric mucosal lesion (GML) model groups. The GML rats were modeled as stress-induced acute gastric mucosal lesions by water-immersion and restrained stress methods [19]. Before modeling, the experimental rats were fasted for 24 h and had free access to water only. Rats were fixed on boards and were immersed vertically in a homeostatic bath at 23 ± 1°C for 10 h, with the liquid surface up to the level of the xiphoid process of the sternum. Biopsy samples of the gastric mucosa were then taken and placed in phosphate-buffered 10% formalin for histological assessment of GML model. After sample dehydration, the biopsies embedded in wax were sectioned at 5 *μ*m and stained with hematoxylin and eosin for histopathological examination by light microscopy.

### 2.2. EA Treatment

After modelling, EA treatment was conducted on either the stomach meridian (SM) or the gallbladder meridian (GM) acupoints for both control and GML rats. Therefore, 6 groups of samples were obtained, that is, control group with no EA treatment (*n* = 8), controls with EA treatment on the SM acupoints (Ctrl-SM group, *n* = 8), controls with EA treatment on the GM acupoints (Ctrl-GM group, *n* = 8), GML model group with no EA treatment (GML group, *n* = 7), GML rats with EA treatment on the SM acupoints (GML-SM group, *n* = 8), and GML rats with EA treatment on GM acupoints (GML-GM group, *n* = 7). For SM acupoints, three acupoints including* Sibai* (ST 2),* Liangmen* (ST 21), and* Zusanli* (ST 36) were selected which represent acupoints of different level (head, trunk, and limb). On the other hand,* Yangbai* (GB 14),* Riyue* (GB 24), and* Yanglingquan* (GB 34) in the same horizontal level were selected for GM acupoints.

The locations for both SM and GM acupoints were determined according to Government Channel and Points Standard GB12346-90 of China and “The Veterinary Acupuncture of China.” Two-channel electrical stimulations were performed via the four stainless-steel acupuncture needles of 0.25 mm in diameter being inserted into the acupoints with a pulse generator (Model G6805-II; Qingdao Xinsheng Medical Instrument Factory, Shandong, China). The electrical stimuli consisted of intermittent and irregular wave (intermittent wave: 4 Hz, irregular wave: 50 Hz) with voltage ranging from 2 to 4 V. The electrical intensity was just strong enough to elicit slight twitches of the hind limbs. Animals were treated for 30 min/day with a seven-day course.

### 2.3. Bio-Sample Collection and NMR Experiments

After the seven-day EA treatment course, 24 hours' urine samples of all rats were collected from metabolism cage. Whole blood was drawn from carotid arteries using a catheter and left to clot at room temperature for 1 h. Samples were centrifuged at 10 000 rpm for 10 min at 4°C to remove particulate contaminants and then stored at −80°C for NMR experiments.

For urine samples, a volume of 300 *μ*L was mixed with 300 *μ*L phosphate buffer solution (1.5 M K_2_HPO_4_/NaH_2_PO_4_, pH 7.4, 99.9% D_2_O) to reduce pH variation across samples. 0.3 mM TSP (3-(trimethylsilyl) propionic-2,2,3,3-d4 acid sodium salt) was used as an internal reference standard at *δ* 0.0. For serum samples, a volume of 400 *μ*L was mixed with 200 *μ*L phosphate buffer solution (90 mM K_2_HPO_4_/NaH_2_PO_4_, pH 7.4, 99.9% D_2_O) without TSP. Then the mixture was transferred to centrifugal tube and centrifuged at 10 000 rpm for 10 min at 4°C. 500 *μ*L of supernatant was piped into 5 mm NMR tube for NMR experiment.

The analyses of the urine and serum samples were performed on Varian NMR system 500 MHz spectrometer equipped with a triple resonance probe. The experimental temperature was set to 298 K and the 90° pulse length was calibrated individually for each sample. For the urine samples, a conventional presaturation pulse sequence for solvent suppression based on the 1D version of NOESY pulse sequence known as NOESYPR (Nuclear Overhauser Effect Spectroscopy with Presaturation, delay-90°-*t*_1_-90°-*t*_*m*_-90°-acquisition) was used [20], where the *t*_1_ represented the first increment in the NOESY experiment and was set to 2 *μ*s; a weak irradiation on water signal was applied to suppress solvent during the mixing time *t*_*m*_ of 120 ms and recycle delay of 2 s. For the serum samples, an additional Carr-Purcell-Meiboom-Gill (CPMG) spin-echo pulse train [21] was incorporated into the NOESYPR sequence with a relaxation time (2*nτ*) of 100 ms and an echo time (*τ*) of 250 *μ*s. A total of 64 scans with a spectral width of 10 kHz were collected for every NMR spectrum. All the signals were zero filled to 32 k before Fourier transformation (FT). The assignments of endogenous metabolites in the urine and serum ^1^H NMR spectra were made with reference to published data [22] and HMDB database (http://www.hmdb.ca/).

### 2.4. Data Preprocessing of ^1^H NMR Spectra

The acquired ^1^H NMR spectra were phased and baseline corrected using MestReNova v.8.1.2 software (Mestrelab Research S.L.). All the spectra were also peak-aligned manually to overcome the peak shift problem [23]. ^1^H NMR spectra were referenced to the internal lactate CH_3_ resonance at 1.33 ppm (for serum spectra) and the single peak of TSP at 0.0 ppm (for urinary spectra). Then, the chemical shift ranges including the resonances from water and urea were manually removed; the peak-free regions were also removed. The spectra over the ranges of 0.5–9.0 ppm for serum samples and 0.5–10.0 ppm for urine samples were binned automatically into regions with fixed width of 0.002 ppm. Prior to statistical analysis, the bucketed data were then normalized by the method of probabilistic quotient normalization [24] to compensate for the differences in overall metabolite concentrations. The NMR spectral data were saved to Microsoft Excel format files and imported into SIMCA-P software (version 12.0.1, Umetrics AB, Umeå, Sweden) for multivariate analysis.

## 3. Results and Discussion

### 3.1. Histological Morphology Examinations

First, the ulcer scores of gastric mucosa for four groups of samples (*i.e.*, control, GML, GML-SM, and GML-GM) were determined based on Guth [25] standards. As shown in Table 1, the ulcer score was the lowest in the control group and the highest in the GML group (*p* < 0.05), suggesting a successful GML model construction. Compared to the GML group, the ulcer scores of the GML-SM group were significantly lower (*p* < 0.05) which indicated EA treatment had conferred healing effect on gastric ulcer. Interestingly, the GML-GM group showed improvement in ulcer scores (*p* < 0.05) but to a lesser extent as compared to the GML-SM group (*p* < 0.05).

**Figure 1:**
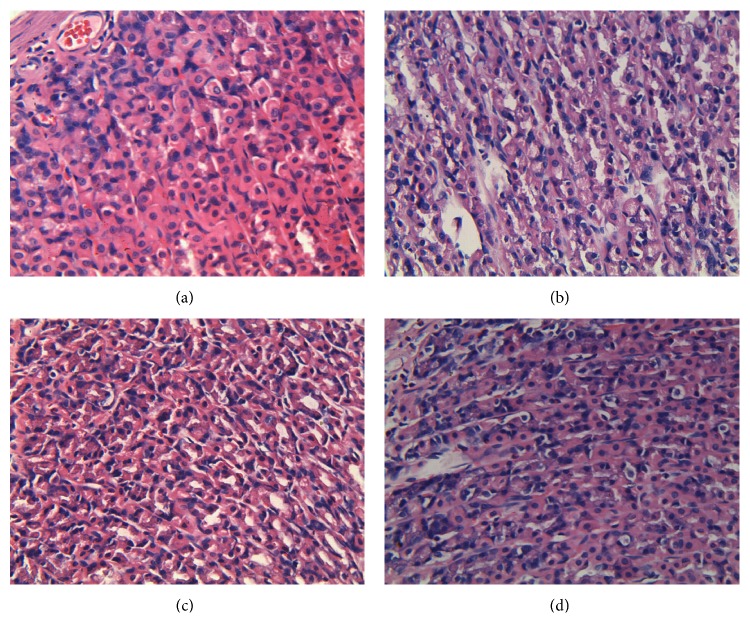
Photomicrographs of representative sections of gastric mucosal tissues from groups of control (a), GML (b), GML-SM (c), and GML-GM (d). The tissue sections were stained with hematoxylin-eosin and observed under a 400x microscope.

Microscopic examinations were conducted on the gastric mucosal tissues from the controls, GML model, and GML groups with EA treatments (Figure 1). The control rats preserved the integrity of gastric mucosal enterocyte structure with obvious staining, well-arranged cells, clearly visible cytoplasm, and abundant capillary. The submucosa and muscularis were continuous without inflammatory cellular infiltration. The disruption of gastric mucosal structure and the difficulty of staining were found for the GML model group (Figure 1(b)). For the GML group, there were many necrotic cells in the glands of gastric mucous where cells were arrayed in disordered state with dissolved nucleus and indistinct cytoplasm. Obvious edema in cells and significant inflammatory cellular infiltration were visible at the same time. While in Figure 1(c), EA treatment on the SM acupoints of GML rats rendered the epithelial cells of gastric mucosa with a fairly complete structure and neat arrangement. The nucleus and cytoplasm were distinct with only a few cellular swelling, inflammatory cells and erythrocytes. The gastric glands had almost healed indicating the effectiveness of EA treatment on SM acupoints. On the other hand, EA treatment on GM acupoints conferred a partial healing effect (Figure 1(d)), as the epithelial area of gastric mucosa and a part of gastric glands were still defective. This was evident by irregular arrangement of cells, indistinct nucleus and cytoplasm, cellular swelling, and considerable inflammatory cells and erythrocytes for the GML-GM group.

### 3.2. Qualitative Analysis by PLS-DA Model

**Figure 2:**
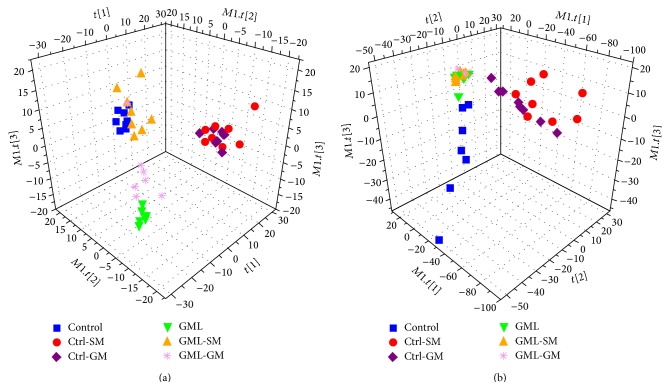
PLS-DA models derived from NMR datasets. (a) Serum score plot (*R* ^2^ *X* = 49.8%, *R* ^2^ *Y* = 63.9%, *Q* ^2^ = 49.4%) and (b) urine score plot (*R* ^2^ *X* = 69.8%, *R* ^2^ *Y* = 59.1%, *Q* ^2^ = 39.8%).

The partial least squares–discriminate analysis (PLS-DA), a supervised pattern recognition technique, was applied to examine group discrimination for both serum and urine datasets. Score plots of the first three components of PLS-DA models were shown in Figure 2, and the results of permutation tests (data not shown) suggested both models were valid and robust.

In the PLS-DA score plots, each point represents a sample. A close spatial distance between points indicates, to some extent, that they are metabolic similar, and vice versa. For both serum and urine datasets, clear separation can be seen in PLS-DA score plots for the control and GML groups, indicating significant metabolic variations between these two groups. GML samples with EA treatment are placed in between control and GML samples in the serum scores plot (Figure 2(a)), suggesting some GML-induced metabolic perturbations are reversed towards healthy state following EA treatment. Relative to GML-GM group, the GML-SM group samples locate closer to the control group which suggests more significant recovery from disease state by acupuncturing the SM acupoints. This is consistent with findings on ulcer scores.

For urinary dataset, PLS-DA score plot (Figure 2(b)) shows clustering of samples collected from all three groups of rats with gastric mucosal lesion (i.e., GML, GML-SM, and GML-GM). EA treatment appears to have mild effects on urinary metabolic profiling that the GML, GML-SM, and GML-GM groups are considerably overlapping with each other. Data points of the control, Ctrl-SM, and Ctrl-GM groups are more scattered compared to other three groups with distinct intraclass grouping in score plots. For urine metabolome, pathological lesions in GML seem to cause main metabolic variation, suggesting that urine metabolome analysis may be effective for GML detection.

### 3.3. Variation Decomposition Using ANOVA

There are two main stimuli (or said factors) induced by experimental design for the experiments rats, that is, disease (GML) stimulus and electroacupuncture stimulus. Both disease and EA can cause metabolic variation in the rats. To distinguish disease- or EA-induced metabolic variations, we used two-way analysis of variance (ANOVA) to decompose the data matrix into a series of submatrices, for example, the effect of disease stimulus, the effect of EA stimulus, the effect of interaction between disease and EA stimuli, and the residuals [26, 27]. These submatrices all had the same dimensionality and followed the general linear model where the percentage of variation in each matrix was displayed in Table 2.

Data variation caused by disease and EA factors was 16.5% and 9.2% of total variation in serum dataset, respectively, while residuals including individual differences, experimental errors, and other unknown errors exceeded 50% of total variation. The 14.4% of total variation for the interaction between two main effects showed that rats under the healthy or GML states may have different metabolic response to EA stimulus. In urine dataset, the variations of disease and EA factors are both higher at 39.1% and 11.6%, respectively, while the percentage of residual variation was 39.4%. It is suggested that there is more sensitive metabolic response to pathological and EA stimuli in urine compared to in serum. Generally, data variation of disease factor is larger than EA factor indicating more significant metabolic changes induced by the pathological lesions.

Following variance decomposition, we then examined the metabolic variation induced by one main factor at a time. For example, disease-related dataset was constructed by adding disease-induced variation and residuals matrix together while discarding the EA and disease-EA interaction matrix. This operation is useful to distinguish metabolic variations due to either GML or EA treatment.

### 3.4. Identification of Metabolic Changes for GML Disease

To get a deeper understanding of the perturbation in metabolome upon GML modeling, orthogonal partial least square discrimination analysis (OPLS-DA), a supervised method, was constructed on the NMR spectra of GML-related dataset that consisted of the control and GML groups after removing the effect of EA and disease-EA interaction factors.

**Figure 3:**
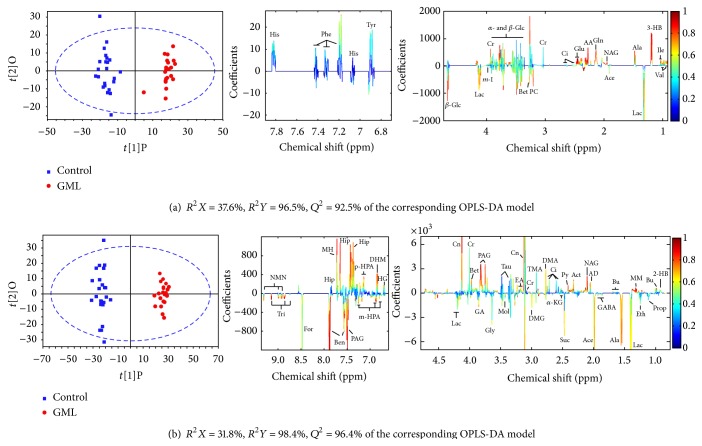
OPLS-DA score plots (left panel) and the corresponding coefficient loading plots (middle and right panels) derived from NMR data of serum (a) and urine (b) obtained from GML-related dataset (keys: 2-HB: 2-hydroxybutyrate; 3-HB: 3-hydroxybutyrate; AA: acetoacetate; ACh: acetylcholine; Ace: acetate; Act: acetone; AD: acetamide; Ala: alanine; Ben: benzoate; Bet: betaine; Bu: butyrate; Ci: citrate; Cn: creatinine; Cr: creatine; DHM: 3,4-dihydroxymandelate; DMA: dimethylamine; DMG: N,N-dimethylglycine; EA: ethanolamine; Eth: ethanol; For: formate; GA: guanidinoacetate; GABA: gamma-aminobutyrate; Gln: glutamine; Gly: glycine; HG: homogentisate; Hip: hippurate; Ile: isoleucine; *α*-KG: *α*-ketoglutarate; Lac: lactate; m-HPA: meta-hydroxyphenylacetate; m-I: myo-inositol; MM: methylmalonate; MH: methylhistidine; MN: 1-methylnicotinamide; Mol: methanol; NAG: N-acetylglutamate; NMN: N-methylnicotinamide; p-HPA: para-hydroxyphenylacetate; PAG: phenylacetylglycine; PC: phosphocholine: Py: pyruvate; Suc: succinate; Tau: taurine; TMA: trimethylamine; Tri: trigonelline; Val: Valine; *α*-Glc: *α*-glucose; *β*-Glc: *β*-glucose).

The OPLS-DA score plots (left panel in Figure 3) show a clear group separation for serum and urine metabolomes. The loading plots (middle and right panels in Figure 3) offer an insight into the characteristic differential metabolites. The loading plots were color-coded by the absolute values of correlation coefficients (|*r*|) between the variable's intensities and the samples' class attributes *Y*. A hot color corresponds to the metabolite with major difference between two selected groups. High explained variation *R* ^2^ *Y* and prediction capability *Q* ^2^ (Figure 3) of the OPLS-DA models suggest that all the models are valid and robust, implying that metabolic differences induced by pathological lesions played an important role in group separation.

As shown in Figure 3, the metabolites marked on the positive direction of the loading plots represent that these metabolite levels are higher in the groups located in the positive direction of first component in the score plots and vice versa. Loadings from identified noise regions in NMR spectra were set as zero manually to avoid baseline distortion. Compared to the control rats, serum metabolome of GML rats was highlighted with high level of isoleucine, 3-hydroxybutyrate (3-HB), N-acetylglutamate (NAG), glutamine, and acetoacetate, together with relatively low level of lactate and betaine (all *p* < 0.05). In urinary data analysis, increased level of methylmalonate (MM), NAG, creatinine, 3,4-dihydroxymandelate (DHM), and hippurate and reduced level of alanine, succinate, and benzoate were found in the GML rats, as compared to the control rats (all *p* < 0.05).

In order to examine the significance of the metabolic perturbation in serum and urine NMR spectra, the means and standard deviations of spectral variables representing selected differential metabolites were calculated for the controls, GML, GML-SM, and GML-GM groups. The significance of intergroup differences was estimated by unpaired *t*-test with a threshold of *p* < 0.05. Following EA treatment on the SM acupoints, NAG, glutamine, and betaine in serum, and alanine, succinate, MM, creatinine, and hippurate in urine were found partially reversed from the GML state (all *p* < 0.05). These biochemical changes were then interpreted with a combination of pathway analysis [28, 29] and published literatures.

The gastric mucosal lesions in rats are characterized by enhanced lipid peroxidation, decreased blood microcirculation of membrane, and increased anaerobic glycolysis in gastric mucosal cells. The upregulation of lactate in serum could be associated with imbalance of its production and clearance induced by cellular anaerobic glycolysis. Previously, a decrease in level of succinate has been recognized as a biomarker for severe gastric ulceration in stomach tissue extracts and serum [30, 31]. Succinate is known to be an intermediate in the citric acid cycle and is catalyzed to form fumarate by the succinate dehydrogenase (SDH) in the mitochondria. The downregulated excretion of succinate is correlated to the disturbance of citric acid cycle. Meanwhile, the marked increase of PAG also reveals a disordered fatty acid metabolism in GML rats. Notably, urinary acyl glycine has been used in the diagnosis of mitochondrial fatty acid *β*-oxidation disorders [32].

As compared to the controls, we observed an increased concentration of hippurate and a decreased benzoate level in the urine of the GML rats (both *p* < 0.05). The conversion of benzoate to hippurate occurs in mitochondrial and is presumably catalyzed by benzoyl-CoA synthetase and benzoyl-CoA/glycine N-acyltransferase [33, 34]. In this study, abnormalities of urinary benzoate and hippurate are generally believed to be associated with microflora dysbiosis because of the destruction of gastric mucosa. Furthermore, elevated levels of 3-HB and acetoacetate were also found in the GML rats. The excess production of acetoacetate in liver has been reported under certain conditions of poor metabolism such as diabetes mellitus leading to excessive fatty acid breakdown. It is then partially converted to acetone by decarboxylation and excreted either in urine or in serum. Like the acetoacetate and acetone, levels of 3-HB in serum and urine are also raised in ketosis. Given that 3-HB is a final product and marker of fatty acid *β*-oxidation in mitochondria, elevated levels of 3-HB and acetoacetate may suggest an impairment of mitochondrial activity in GML rats.

Betaine serves as organic osmolytes in biological systems. It plays a physiological role in endogenous mucosal protection. Decrease betaine concentration (*p* < 0.05) found in the GML rats indicated a dysfunction of gastric mucosal due to disease modelling. Isoleucine, one of branched chain amino acids (BCAA), is found to be increased in serum from pancreatic cancer patients [35]. It can be speculated that abnormal metabolism of isoleucine is connected to the digestive system disorder. Furthermore, the concentration of alanine is influenced when it was produced in the body from pyruvate and BCAA. MM is a malonic acid derivative which is a vital intermediate in the metabolism of fat and protein. Abnormalities in MM metabolism denoted a block in the enzymatic conversion of methylmalonyl CoA to succinyl CoA in the GML rats.

### 3.5. Identification of Metabolic Changes for EA Treatment

In TCM theory, acupoints are the specific sites that reflect and adjust the changes of visceral function. They play an important role in diagnosing and treating pathological lesions of organs. Stimulating the corresponding acupoints would dredge channel of Qi (vital energy) and blood to achieve efficacy of prevention and treatment of disease and to stay healthy. Although the benefits of acupuncture have been confirmed by long-term clinical practice, their physiological mechanism is still unknown. Here the metabolic response to electroacupuncturing on acupoints was evaluated from metabolomics perspective based on the urine and serum metabolome by multivariate statistical analysis. In order to discriminate the biochemical variations after stimulating different acupoints, the stomach meridian (SM) acupoints were picked up as a functional point to stomach with the gallbladder meridian (GM) acupoints chosen as a control acupoint.

**Figure 4:**
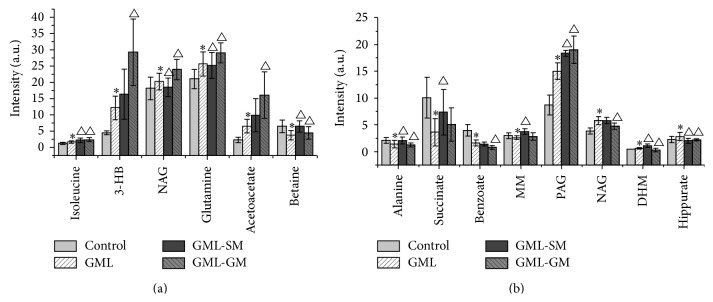
Quantitative changes of serum (a) and urine (b) metabolome from the control, GML group, and GML-SM and GML-GM groups. ∗ indicates *p* < 0.05 statistical significance relative to control group; △ indicates *p* < 0.05 statistical significance relative to GML group after electroacupuncture in SM or GM acupoints of GML rats.

**Figure 5:**
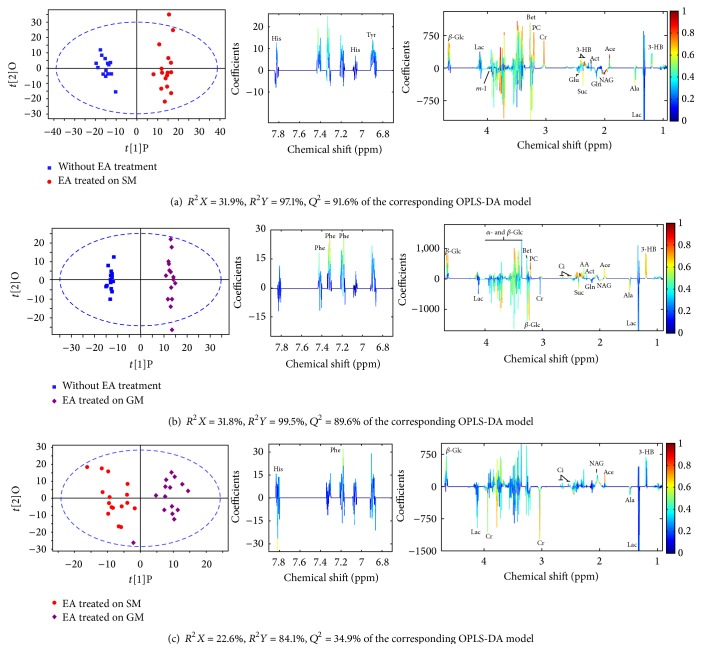
OPLS-DA score plots (left panel) and the corresponding loading plots (middle and right panels) derived from NMR data of serum obtained from two-group comparison focusing on the metabolic difference due to EA treatment.

**Figure 6:**
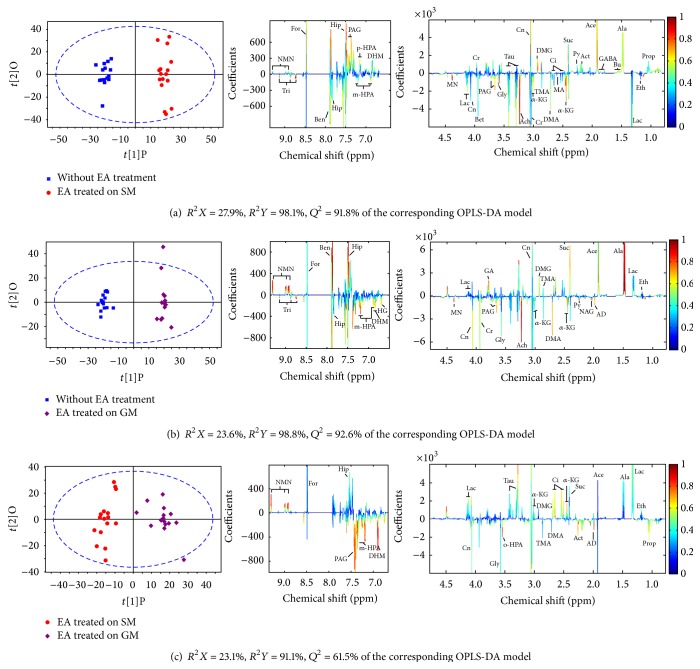
OPLS-DA score plots (left panel) and the corresponding loading plots (middle and right panels) derived from NMR data of urine obtained from pairwise EA-related datasets.

Then, OPLS-DA models were built for two-group comparison between GML rats with or without EA treatments (i.e., EA treatment on SM, EA treatment on GM, and with no EA treatment) (Figures 5 and 6). In the comparison, variation due to the GML factor was removed using ANOVA variance decomposition. An eightfold cross-validation and random permutation tests were carried out on the corresponding PLS-DA models to validate the reliability and robustness of models. Clear separation of two groups was observed on all three score plots of OPLS-DA model (left panel in Figures 5 and 6) demonstrating intrinsic metabolic differences between groups. The similarities and differences in serum and urine metabolome were examined by inspection of loading plots on the middle and right panels of Figures 5 and 6. Compared to the group without EA treatment, higher level of acetate, 3-HB, creatine, phosphocholine, N,N-dimethylglycine (DMG), and hippurate, phenylacetylglycine (PAG) and lower level of NAG, acetylcholine, *α*-ketoglutarate, 1-methylnicotinamide (MN), and trigonelline were found in rats with EA treated on SM acupoints (all *p* < 0.05), while in rats with EA treated on GM acupoints, the set of perturbed metabolites was found quite similar to that from EA treatment on SM acupoints, suggesting that these metabolic perturbations can be associated with the improvement in ulcer scores for both groups. Notable exception included alanine and N-methylnicotinamide (NMN) which were particularly highlighted in urine of GML-SM group, as compared to rats without EA treatment. The direct comparison of EA treated on SM and GM group showed significant differences mainly in aromatic region of ^1^H-NMR spectra, that is, increased PAG, DHM, and m-HPA concentrations and decreased NMN concentration in the urine of GML-SM rats (all *p* < 0.05). These metabolic changes may contribute to a further improvement in ulcer score for GML-SM group, as compared with the GML-GM group. Although the urinary concentration of PAG from GML-SM rats in Figure 4 seemed lower than that from GML-GM rats, this result was obtained from the original dataset without ANOVA-PCA analysis that may be interfered by GML factor. The variations of m-HPA and NMN were not observed in Figure 4 which could be attributed to their significance level lower than setting value.

Acupuncture treatment involves the insertion of thin needles into the skin and underlying muscle layer. Thus, this procedure may stimulate the somatic afferent nerves of the skin and muscles [36]. Following EA treatment on SM acupoints, a potent vasodilator reaction of gastric vessels may be induced with blood perfusion in the moderate and small vessels and microcirculation increased in certain degrees, and this increase may offset the natural decrease of blood flow [37]. Furthermore, acupuncture at the* Zusanli *point has been proved to increase the secretion of motilin and cholecystokinin.

More detailed analysis of pathways and network influenced by EA treatment was performed by the online MetaboAnalyst toolbox and interpreted using published findings. Acetylcholine is an important excitatory neurotransmitter at many sites in the central nervous system. Various nicotinic and muscarinic acetylcholine receptors are present in both afferent nerve endings and glomus cell. The activity of acetylcholine esterase in the intramural nerve plexuses of the gastrointestinal tract was enhanced after acupuncture at the* Zusanli* point [37]. Therefore, the variation of acetylcholine might be connected with needles-stimulated somatic afferent nerves. While acetylcholine is hydrolyzed to acetate and choline by acetylcholinesterase, this may explain a higher acetate concentration in rats with EA treatment.

As mentioned above, the concentrations of 3-HB and hippurate were correlated with the function of mitochondria in fatty acid *β*-oxidation and energy metabolism. The elevation of these two metabolites suggested a functional regulation of mitochondria. In addition, *α*-KG is known as a key intermediate in citric acid cycle that reacts with ammonia to form glutamate and then glutamine. Glutamate together with acetyl-CoA would further biosynthesize NAG by the enzyme N-acetylglutamate synthase. Although the changes of glutamate and glutamine were unidentifiable based on present NMR dataset, they were reported to be activated by acupuncture treatment at “*Zusanli*” acupoints [38]. Glutamate serves as the most abundant fast excitatory neurotransmitter in the mammalian nervous system and is probably perturbed by EA treatment. Thus the metabolic pathways of *α*-KG + NADH → glutamate + NAD^+^ and glutamate → NAG may be both influenced through EA treatment.

The differences between SM and GM acupoints stimulation were denoted with increased PAG, DHM, and m-HPA as well as decreased NMN concentration in GML-SM rats. The upregulation of PAG reveals a significant stimulation of the activity of microbiota by EA treated on SM acupoints. Although the fact that the biochemical basis of three other metabolites contributed to the group discriminations remains unclear, it provides an insight into the curative differences upon EA treatment on different acupoints.

## 4. Conclusion

In this study, ^1^H NMR-based metabolomics was applied to investigate the effect of EA therapy on the urine and serum metabolome from a gastric mucosal lesion rat model. Supervised multivariate methods including PLS-DA and OPLS-DA were combined with univariate analysis to identify differential metabolites that can be associated with the healing effect of EA treatment. Clear metabolic differences were observed between GML and control groups and related metabolic pathways were interpreted using an online metabolic network analysis toolbox. By comparing the endogenous metabolites from GML and GML-SM groups, the perturbed pathways were partially reversed towards normal state via EA treated on SM acupoints. Further investigation of the metabolic variations on EA stimulated on functional and nonfunctional acupoints showed that affected metabolite composition was highly similar except for increased PAG, DHM, and m-HPA concentrations and decreased NMN concentration in the urine of GML-SM rats. These results indicated that comprehensive metabolomics approach is a potential tool to explore the biochemical basis and metabolic mechanism of acupuncture treatment. With the further development of metabolomics with multianalytical techniques integration, it may bridge Western and traditional Chinese medicine and promote the modernization of TCM research.

## Figures and Tables

**Table 1:** The ulcer scores of gastric mucosa from four groups ($\bar{x}$ ± *sd*⁡).

Group	Ulcer score
Control	6.2 ± 0.5
GML	35.6 ± 0.9^∗^
GML-GM	24.4 ± 0.7^∗△^
GML-SM	17.2 ± 0.7^∗△▲^

The lesions are scored as follows: (petechial lesions) = 1, (erosions <1 mm) = 2, (erosions between 1 and 2 mm) = 3, (erosions between 2 and 4 mm) = 4, and (erosions greater than 4 mm) = 5. The partial scores were then summed to obtain the final total lesion score for that rat. ∗ indicates *p* < 0.05 statistical significance relative to control group; △ indicates *p* < 0.05 statistical significance relative to GML group; ▲ indicates *p* < 0.05 statistical significance relative to GML-GM group with respect to *t*-test.

**Table 2:** Percentage of data variation in original data matrix.

Dataset	Disease	EA	Disease-EA interaction	Residual	Total
Serum	16.5%	9.2%	14.4%	59.9%	100.0%
Urine	39.1%	11.6%	9.9%	39.4%	100.0%
